# Medical Society of the State of New York

**Published:** 1893-02

**Authors:** 


### Society Meetings.

### MEDICAL SOCIETY OF THE STATE OF NEW YORK.

*Eighty-seventh Annual Meeting.*

The annual meeting will be held in the City Hall, at Albany, Tuesday, Wednesday, and Thursday, February 7, 8, and 9, 1893, commencing at 9.15 a. m. on Tuesday, and ending at 1 p. m. on Thursday. A cordial invitation is extended to all members of the County societies.

PROVISIONAL PROGRAMME.

*Tuesday, February* 7, 1893.—*Morning Session.*—9.15—Call to Order; President's Inaugural Address; Executive Business. 10.30—Scientific Business: Landon Carter Gray, M. D., New York, The Relation, in the Male and Female, of Genital Disease to Mental and Nervous Affections. 11.00—Special Order for the Morning: Topic—Epilepsy. (1.) The Epileptic Interval; Its Phenomena and their Importance to Treatment, William Browning, M. D., Brooklyn. (2.) Reflex Disturbances in the Causation of Epilepsy, William C. Krauss, M. D., Buffalo. (3.) Mental Epilepsy, J. Montgomery Mosher, M. D., St. Lawrence State Hospital for the Insane, Ogdensburg. (4.) The Development of Epilepsy after Traumatic Injury to the Skull, B. Sachs, M. D., New York. 12.45—Adjournment.

*Afternoon Session.*—2.15—Call to Order; Executive Business. 2.45—Scientific Business: R. C. M. Page, M. D., New York, The Treatment of Uremic Convulsions; J. L. Kortright, M. D., Brooklyn, The Registration of Midwives. 3.30—Special Order for the Afternoon: Topic—The Relative Value of Certain Obstetrical Operations. (1.) General Review of the Operations to be Discussed, by Dr. Egbert H. Grandin, of New York. (2.) The Limitations of Embryotomy, by Dr. N. Clifton Edgar, New York. (3.) The Limitations of the Cesarean

Section, by Dr. Robert A. Murray, New York. (4.) The Anatomical Limitations of Symphysiotomy, by Dr. J. E. Kelly, New York. (5.) The Clinical Limitations of Symphysiotomy, by Dr. Charles Jewett, Brooklyn. General Discussion, to be participated in by Drs. E. P. Davis, Philadelphia; H. A. Kelly, Baltimore; H. C. Coe, New York; Reynolds, Boston. 6.00—Adjournment.

*Evening Session.*—7.45—Howard A. Kelly, M. D., Baltimore, Practical Antisepsis and Asepsis, with Stereopticon Demonstrations (by invitation). 9.00—Joseph H. Hunt, M. D., Brooklyn, Epitaphs from the Tombstones of Medical History, with Stereopticon Projections of Many Old and Rare Medical Portraits.

*Wednesday, February* 8, 1893.—*Morning Session.*—9.15—Call to Order; Executive Business. 9.30—Scientific Business: Topic—The Management of Suppuration Complicating Tuberculous Disease of the Bones and Joints. Papers by Drs. V. P. Gibney, of New York; Roswell Park, of Buffalo; Henry Ling Taylor, of New York; and Louis A. Weigel, of Rochester. 10.30—Special Order for the Morning: Topic—The Present State of Knowledge as to Carcinoma. (1.) The Pathology of Carcinoma, H. C. Coe, M. D., New York. (2.) The Etiology of Carcinoma, Roswell Park, M. D., Buffalo. (3.) The Value of Internal Medication in the Treatment of Carcinoma, Jarvis S. Wight, M. D., Brooklyn. (4.) The Results Obtainable from the Use of Analine Products in Carcinoma, Willy Meyer, M. D., New York. (5.) Caustics in the Treatment of Carcinoma, Daniel Lewis, M. D., New York. (6.) The Knife in the Treatment of Carcinoma, N. Jacobson, M. D., Syracuse. Discussion to be opened by Dr. George R. Fowler, of Brooklyn. 12.45—Adjournment.

*Afternoon Session.*—2.15—Call to Order; Executive Business. 2.30—Scientific Business: Herman Mynter, M. D., Buffalo, Tuberculous Epididymitis. 3.00—Special Order for the Afternoon: Topic—Newer Methods of Diagnosis and Treatment of Stomach and Intestinal Diseases. (1.) The Practical Value of the Newer Methods of Examination in the Diseases of the Stomach, with a Consideration of the Indications Given for Diet and Treatment by such Examinations, by Henry L. Elsner, M. D., Syracuse. (2.) The Methods of Obtaining and Examining the Stomach Contents in Disease for Purposes of Diagnosis, by J. Fuhs, M. D., Brooklyn. (3.) The Disturbances of the Motor Function of the Stomach; their Diagnosis, Symptoms, and Treatment, by C. G. Stockton, M. D., Buffalo. (4.) The Physiological Effect of Electricity in the Stomach, the Indications for its Administration and Use in Gastric Disease, and the Methods of Using the Same, by Max. Einhorn, M. D., New York.

*Evening Session.*—8.00—Anniversary Address by the President in the Senate chamber. Topic—The Evolution of the American Surgeon. 9.30—Annual dinner of the Society at the Delavan House.

*Thursday, February* 9, 1893.—*Morning Session.*—9.15—Call to Order ; Executive Business. 9.30—Scientific Business : J. S. Cooley, M. D., Glen Cove, Report of a Case of Severe Abdominal Injury Terminating in Recovery ; Alex. Dallas, M. D., New York, The Treatment of Inguinal Hernia ; Andrew F. Currier, M. D., New York, Certain Types of Septicemia Resulting from Abortion ; Wm. W. Potter, M. D., Buffalo, Puerperal Sepsis, Its Prevention and Cure ; W. Franklin Chappell, M. D., New York, Hoarseness ; Nelson G. Richmond, M. D., Fredonia, The Diagnosis and Nomenclature of Fevers ; Wm. F. Mittendorf, M. D., New York, Congenital Opacities of the Lens ; W. Freudenthal, M. D., New York, Is Stoerk's Blenorrhea and Laryngitis Sicca One and the Same Disease ?

The Committee on Credentials will meet at the Delavan House Monday evening, when members and delegates can register. The Committee consists of Dr. Walter B. Chase, of Brooklyn ; Dr. Charles M. Culver, of Albany ; and Dr. J. P. Creveling, of Auburn.

Communications regarding papers should be addressed to the Business Committee : Dr. Seneca D. Powell, 12 W. Fortieth street, New York ; Dr. William Maddren, 1 Hanson place, Brooklyn ; Dr. John O. Roe, 28 N. Clinton street, Rochester.

